# Variation in KSHV prevalence between geographically proximate locations in Uganda

**DOI:** 10.1186/s13027-020-00313-8

**Published:** 2020-07-23

**Authors:** Angela Nalwoga, Emily L. Webb, Claudios Muserere, Belinda Chihota, Wendell Miley, Nazzarena Labo, Alison Elliott, Stephen Cose, Denise Whitby, Robert Newton

**Affiliations:** 1 MRC/UVRI and LSHTM Uganda Research Unit, Entebbe, Uganda; 2 Department of Immunology and Microbiology, University of Colorado, Anschutz Medical Campus, Aurora, CO USA; 3 London School of Hygiene & Tropical Medicine, London, UK; 4 Viral Oncology Section, AIDS and Cancer Virus Program, Leidos Biomedical Research, Inc., Frederick National Laboratory for Cancer Research, Frederick, MD USA; 5 University of York, York, UK

**Keywords:** KSHV, Uganda, Seroprevalence, Various populations

## Abstract

Kaposi’s sarcoma-associated herpesvirus (KSHV) transmission within endemic areas may vary. KSHV seroprevalence has been studied by different groups of researchers using different methods, making it difficult to make direct comparisons. Here we show results on KSHV seroprevalence using the same laboratory method from four different but geographically proximate populations in Uganda.

Blood samples from the urban Entebbe Mother and Baby Study (EMaBS), the rural General Population Cohort (GPC), the fishing community Lake Victoria Island Intervention Study on Worms and Allergy related Diseases (LaVIISWA) and the high-risk sexual behaviour Good Health for Women Project (GHWP), were tested for IgG antibody levels to K8.1 and ORF73 recombinant proteins using ELISA.

All adult participants of the EMaBS study and the GHWP were women, while the GPC (54% female) and LaVIISWA (52% female) studies had both males and females. EMaBS children were all 5 years of age while their mothers were 14 to 47 years of age. GHWP women were 15 to 45 years old, LaVIISWA participants were 1 to 72 years old while GPC participants were 1 to 103 years old. KSHV seropositivity varied in the different populations. In children aged 5 years, EMaBS had the lowest prevalence of 15% followed by GPC at 35% and LaVIISWA at 54%. In adult women, seropositivity varied from 69% (EMaBS) to 80% (LaVIISWA) to 87% (GPC) to 90% (GHWP).

The reasons for the variation in prevalence are unclear but may reflect differences in the prevalence of cofactors between these four geographically proximate populations.

## Key message

We show that KSHV prevalence varies even within geographically proximate locations in Uganda. This variations could be caused by a number of co-factors including malaria, HIV and helminths infections and early age of KSHV infection. The high KSHV prevalence in various populations may be explained by the multiple co-factors that affect KSHV acquisition and transmission.

## Introduction

Uganda has amongst the highest reported incidence of Kaposi’s sarcoma (KS) in the world [1] and the highest reported seroprevalence of the underlying causal agent, Kaposi’s sarcoma-associated herpesvirus (KSHV) [2–4]. Unlike other human herpesviruses, KSHV varies in prevalence widely across the world [5], perhaps reflecting variation in underlying drivers of lytic replication and transmission.

Several risk factors for KSHV have been identified by our group, and others, including infection with HIV, malaria and helminths [2, 3, 6–11]. The distribution of these may vary between regions within the same country leading to differences in the spread of KSHV, even between geographically proximate locations.

In this manuscript, we have examined the seroprevalence of KSHV in four population cohorts in Uganda. These cohorts include an urban population (Entebbe Mother and Baby Study, EMaBS) [12], a land-locked rural population (General Population Cohort, GPC) [13, 14], a rural fishing community population in the islands of Lake Victoria (Lake Victoria Island Intervention Study on Worms and Allergy related Diseases, LaVIISWA) [15], and a high-risk sexual behaviour population recruited in an urban centre, but including highly mobile women who engage in transactional sex (Good Health for Women Project, GHWP) [16].

## Methods

Data on KSHV seropositivity from the EMaBS, the GPC, LaVIISWA and the GHWP were analysed for this manuscript. The study populations are described briefly below and further details have previously been published [12, 13, 15, 16].

**Fig. 1:**
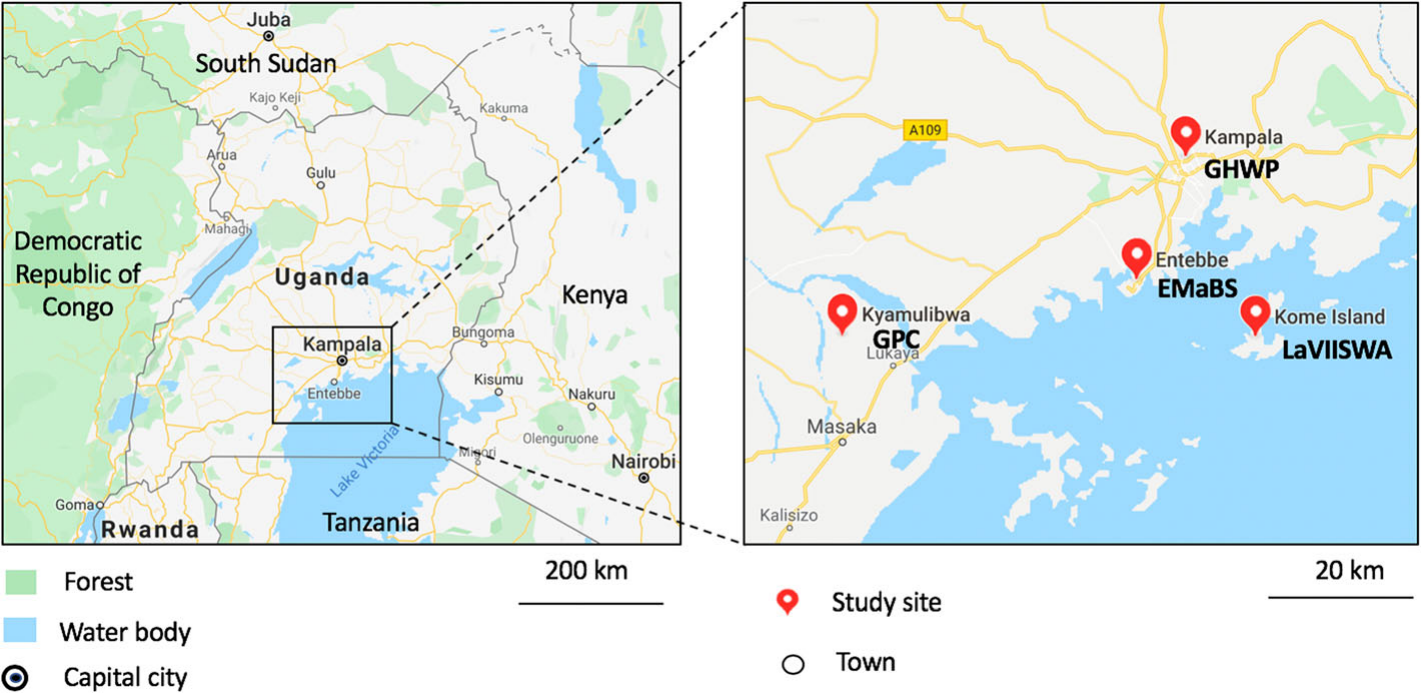
Map showing the location of the study sites. EMaBS-Entebbe Mother and Baby Study, GPC-General Population Cohort, LaVIISWA-Lake Victoria Island Intervention Study on Worms and Allergy related diseases, GHWP-Good Health for Women Project. Drawn from Google maps

The Entebbe Mother and Baby Study (EMaBS; ISRCTN32849447) was a randomised double-blind, placebo-controlled trial of anthelminthic treatment during pregnancy [12, 17–19], designed to investigate the effects of helminth treatment during pregnancy on childhood responses to vaccines and infectious diseases. Pregnant mothers in their second or third trimester attending antenatal care at Entebbe general hospital were recruited between June 2003 and August 2005. A total of 2507 pregnant women from the urban Entebbe municipality and peri-urban Katabi sub-county (Fig. 1) were enrolled in the EMaBS. Demographic and clinical details, blood and stool samples were obtained from the mothers at enrolment and 1 month after delivery, and from children at ages one, two, three, four, five, 6 and 9 years. Using plasma samples from mothers during early postpartum period and 5 years old children, we tested for KSHV antibody responses to determine KSHV seropositivity and its associated risk factors [7, 9, 10, 20]. EMaBS provided a stable urban population relative to the other populations analysed for this paper.

The General Population Cohort (GPC) is located in southwestern Uganda, in Kyamulibwa sub-county of Kalungu district [13, 21] (Fig. 1). The GPC has a population of about 22,000 people scattered across the countryside in 25 adjacent villages [22]. It is a rural community with semi-permanent structures built from locally available materials. The community is relatively stable, homogeneous and young, with 90% of residents aged less than 50 years of age. Only about 13% of the residents attained education beyond primary level [23, 24]. The GPC was established to examine the trends and determinants of the HIV epidemic in rural southwestern Uganda [25, 26]. Biennial population surveys, as well as medical surveys, are conducted at a central hub in each village among all people who have lived in the village for at least 3 months. During each medical survey, blood, biophysical and lifestyle data are collected to determine disease outcomes and risk factors for selected participants [27–29]. To determine KSHV seroprevalence in this area, we have tested plasma samples for KSHV IgG antibodies, collected at various survey rounds, the latest tested being the 24th (2014/2015) and 25th (2016) surveys [2].

The Lake Victoria Island Intervention Study on Worms and Allergy-related diseases (LaVIISWA) was a two-arm open cluster randomised trial (ISRCTN47196031) of intensive versus standard antihelminthic treatment [30]. The trial was carried out in Lake Victoria island fishing villages in Koome sub-county, Mukono district, Uganda (Fig. 1), between September 2012 and August 2016. Kome islands are relatively isolated and rural, consisting of 27 villages with a relatively mobile population of approximately 16,000 people; 26 of the villages participated in the trial with the remaining village used for piloting procedures. The trial aimed to investigate the effect of helminth treatment on allergy-related outcomes in a *Schistosoma mansoni* heavily infected area. Questionnaires were administered to record household features and socio-demographic characteristics [31]. Plasma samples collected from the trial were randomly selected and tested for KSHV IgG antibodies to determine KSHV seropositivity [3].

The Good Health for Women Project (GHWP) is a high-risk sexual behaviour cohort of women who engage in transactional sex. Participants were recruited from a densely populated slum area in southern Kampala, although many were originally from rural areas and are relatively mobile, some moving frequently to find work (Fig. 1). These women work from bars, night clubs, local beer breweries, eating places, lodges and guesthouses known to provide rooms for commercial sex work. Recruitment of over 1000 women into the GHWP occurred in 2008 and three-monthly follow-up was carried out. Details of the study are shown elsewhere [32, 33]. A random sample of sera from 410 women in this cohort was selected to determine KSHV seroprevalence.

All serological analyses were done in the same laboratory at the MRC/UVRI and LSTHM Uganda Research Unit in Entebbe, Uganda. Plasma or serum samples were tested for anti-KSHV IgG antibodies to KSHV-encoded K8.1 and latently associated nuclear antigen (LANA)/ORF73 recombinant proteins using an Enzyme-linked immunosorbent assay (ELISA). Three negative control wells and three positive control wells were included on each plate. The negative controls were used to calculate a cut-off value for each plate. The cut-off value for each plate was the average background-subtracted optical densities (OD) of the three negative control triplicates plus a constant value of 0.75 (for K8.1) or 0.35 (for ORF73/LANA). This procedure has been reported previously [34].

Statistical analysis was carried out using STATA version 13 (StataCorp, College Station, Texas USA). The survey study design of the LaVIISWA trial was not self-weighting (because the number of households selected from each village was fixed, therefore households from smaller villages were more likely to be included in the survey than households from larger villages). To allow for this non-self-weighting design and to ensure that our analyses are representative of the study area, we, therefore, took into account clustering within villages and applied village-level weights for all analyses [35, 36]. A survey weight of one and a unique cluster number were given to each participant from the other studies (EMaBS, GPC and GHWP). Logistic regression (allowing for the survey design) was used to determine associations between the study population and KSHV seropositivity.

## Results

Plasma from 1164 EMaBS women and 1222 children aged 5 years, 403 women aged 15 to 45 years from GHWP, as well as males and females of all ages (7283 (GPC) and 1571 (LaVIISWA)) were analysed (Table 1). HIV prevalence varied in the four different populations with GHWP having the highest prevalence (38%), and the GPC having the lowest (8%) among those aged 14 years and above (Table 1). Other population characteristics of the participants analysed are shown in Table 1.

**Table 1:** Population characteristics

	EMaBS^a^*n* = 2386	GPC^b^*n* = 7283	LaVIISWA^c^*n* = 1571	GHWP^d^*n* = 403
Sample collection years	2003/2006	2014/2016	2015/2016	2008/2009
Age in years, mean (range)	24 (14, 47)^e^	23 (1, 103)	23 (1, 72)	26 (15, 45)
Age groups, years				
1–4		11% (833/7283)	14% (224/1571)	
5	51% (1222/2386)	4% (315/7283)	4% (61/1571)	
6–13		31% (2277/7283)	14% (228/1571)	
14–18	8% (196/2386)	12% (848/7283)	6% (72/1571)	2% (9/402)
19–25	25% (594/2386)	8% (601/7283)	16% (255/1571)	46% (185/402)
26–50	16% (374/2386)	21% (1527/7283)	43% (672/1571)	52% (208/402)
Above 50		12% (882/7283)	4% (59/1571)	
Sex, female	74% (1765/2386)	54% (3925/7283)	52% (770/1571)	100% (403/403)
HIV serostatus				
Children (< 13 years)	2% (22/1221)	1% (36/3126)	2% (6/283)	
Adults (14+ years)	14% (160/1164)	8% (315/3853)	21% (195/946)	38% (153/403)

HIV serostus determine using rapid diagnostic tests ^a^EMaBS-Entebbe Mother and Baby Study, ^b^GPC-General Population Cohort, ^c^LaVIISWA-Lake Victoria Island Intervention Study on Worms and Allergy related diseases, ^d^GHWP-Good Health for Women Project, ^e^Only women from EMaBS, children were all 5 years old

**Fig. 2:**
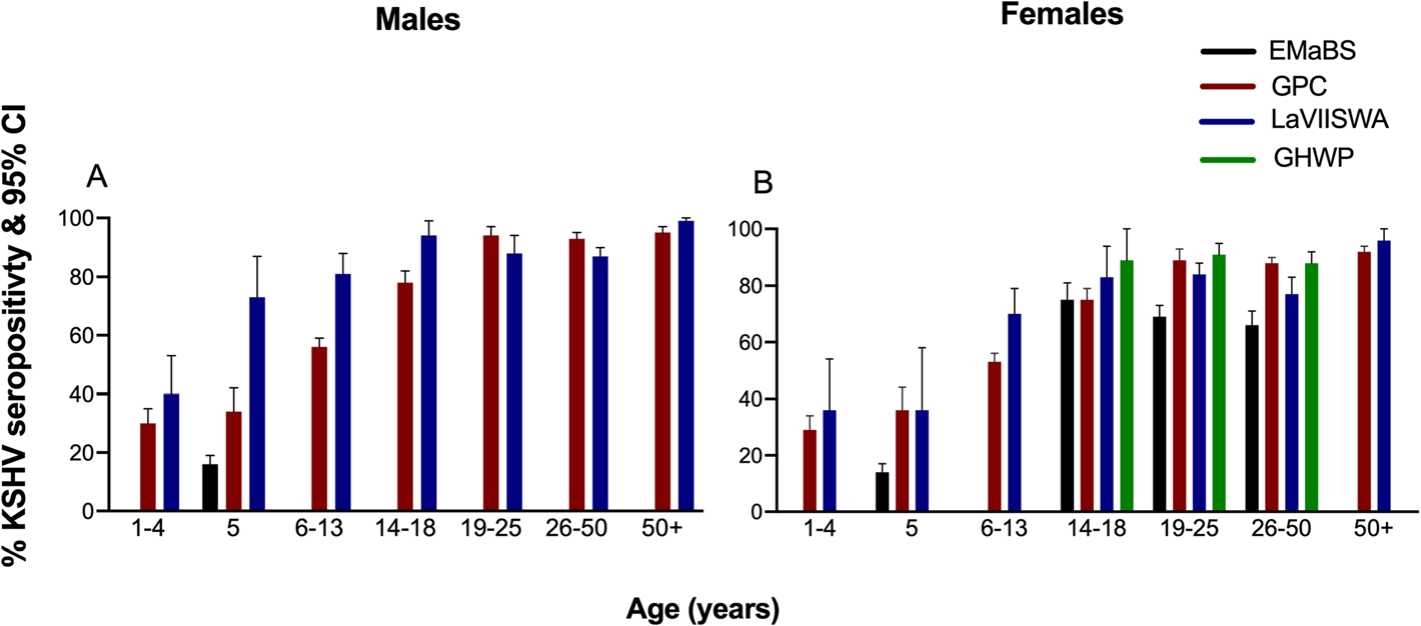
KSHV seropositivity and 95% confidence intervals (CI). KSHV Seropositivity defined as reactivity to either ORF73 or K8.1 proteins. KSHV antibodies were detected using ELISA. Seropositivity and 95% CI were obtained in STATA, allowing for the survey design. Graphs were drawn in Graphpad Prism 8. EMaBS-Entebbe Mother and Baby Study, GPC-General Population Cohort, LaVIISWA-Lake Victoria Island Intervention Study on Worms and Allergy related diseases, GHWP-Good Health for Women Project

First, we looked at KSHV seropositivity in the various age groups from the four different populations. KSHV seropositivity increased with increasing age, (Fig. 2). The urban Entebbe Mother and Baby Study had the lowest seropositivity in all the tested age groups while the GHWP had the highest seropositivity.

After considering the crude pattern of KSHV seropositivity, we then analysed the seropositivity of KSHV in the various study populations adjusting for age, sex and HIV serostatus. A separate statistical analysis of children aged 5 years was done because all EMaBS children included were 5 years old at the time of sampling. In these children, after accounting for the effect of sex and HIV serostatus, the odds of being KSHV seropositive were three times higher in individuals from the GPC (OR = 3.08 (2.31, 4.12), *p* < 0.0001), and seven times higher in LaVIISWA (OR = 7.81 (3.67, 16.62), p < 0.0001), compared to individuals from urban Entebbe (EMaBS); Table 2. This association was maintained when the age of the child was accounted for and all children aged 1 to 13 years were included in the analysis (Table 2). The odds of being KSHV seropositive in women 14 years and over was two times, three times and four times higher in LaVIISWA (OR = 1.72 (1.22, 2.42), *p* = 0.002), GPC (OR = 2.61 (1.94, 3.52), p < 0.0001), and GHWP (OR = 3.90 (2.72, 5.59), p < 0.0001) respectively, compared to women from the EMaBS (Table 3). These differences were maintained when only women of childbearing age (14 to 49 years) were included in the analysis (Table 3). Furthermore, males over 14 years of age had similar odds of being KSHV seropositive if they were from the GPC and LaVIISWA (Table 3). GHWP and EMaBS had no males over 14 years of age.

**Table 2:** Association between KSHV seropositivity or antibody levels and study population. KSHV seropositivity in children

Study	Children 1–13 years		Children 5 years only	
	KSHV seropositivity	*OR (95% CI), *P* value	KSHV seropositivity	**OR (95% CI), P value
EMaBS^a^	15% (184/1222)	1	15% (184/1222)	1
GPC^b^	47% (1597/3425)	3.16 (2.38, 4.19), **< 0.0001**	35% (111/315)	3.08 (2.31, 4.12), **< 0.0001**
LaVIISWA^c^	56% (305/513)	5.79 (3.82, 8.77), **< 0.0001**	54% (37/61)	7.81 (3.67, 16.62), **< 0.0001**

*Adjusted for age group, sex and HIV serostus ** Adjusted for sex and HIV serostatus. *OR: odds ratio. Logistic regression analysis was used for statistical analysis allowing for the survey design for the LaVIISWA trial. ^a^EMaBS-Entebbe Mother and Baby Study, ^b^GPC-General Population Cohort, ^c^LaVIISWA-Lake Victoria Island Intervention Study on Worms and Allergy related diseases

**Table 3:** Association between KSHV seropositivity or antibody levels and study population. KSHV seropositivity in adults

Study	Females 14-49 years		Females 14–103 years		Males 14–103 years	
	KSHV seropositivity	*OR (95% CI), P value	KSHV seropositivity	*OR (95% CI), P value	KSHV seropositivity	*OR (95% CI), P value
EMaBS^a^	69% (806/1164)	1	69% (806/1164)	1		
GPC^b^	85% (1388/1638)	2.60 (1.92, 3.51), **< 0.0001**	87% (1905/2201)	2.61 (1.94, 3.52), **< 0.0001**	90% (1486/1657)	1
LaVIISWA^c^	79% (400/493)	1.69 (1.21, 2.36), **0.002**	80% (425/519)	1.72 (1.22, 2.42), **0.002**	88% (477/539)	0.71 (0.44, 1.15), 0.165
GHWP^d^	90% (361/402)	3.93 (2.74, 5.64), **< 0.0001**	90% (361/402)	3.9 (2.72, 5.59), **< 0.0001**		

* Adjusted for age group and HIV serostatus. *OR: odds ratio. Logistic regression analysis was used for statistical analysis allowing for the survey design for the LaVIISWA trial. ^a^EMaBS-Entebbe Mother and Baby Study, ^b^GPC-General Population Cohort, ^c^LaVIISWA-Lake Victoria Island Intervention Study on Worms and Allergy related diseases, ^d^GHWP-Good Health for Women Project

## Discussion

The worldwide incidence of KS is known to vary greatly, in large part reflecting variation in the prevalence of KSHV, the underlying cause [5]. Less is known about local variation between populations within countries, perhaps because differences between published studies may be attributed to differences between assays and approaches used, rather than reflecting real differences in prevalence. In this paper, we take advantage of results on KSHV seropositivity obtained from various Ugandan populations, tested using the same antigens and carried out by the same group of people from the same laboratory. Additionally, the glycoprotein K8.1 and the latent protein latently associated nuclear antigen (LANA)/ORF73 were used as antigens in the ELISA. These when used in combination have been shown to be very specific and sensitive at detecting HHV8 specific antibodies [34, 37, 38]. In summary, we find that prevalence varies significantly between cohorts recruited from geographically proximate locations.

Here we show that a relatively stable urban population (EMaBS) has the lowest KSHV prevalence compared to the other populations. This may be attributed to the lower occurrence of putative risk factors for KSHV lytic replication and transmission, such as, malaria and helminthic parasites.

The GHWP population was included in the analysis because of it’s uniqueness in comparison to the other populations. It is an urban population with a high HIV risk. It is a very mobile, high-risk sexual behaviour cohort with an HIV seroprevalence of 38%. These women had the highest risk of being KSHV seropositive. This KSHV risk is probably attributable to HIV and behavioural practices, involving salivary exchange. Additionally, many of the women are also highly mobile, moving frequently between the urban setting and more rural areas for work hence increasing their risk of acquiring infections such as KSHV.

After GHWP, the Lake Victoria Island people and the rural GPC people had higher KSHV seroprevalence compared to EMaBS. The highly mobile Island communities of Lake Victoria have high HIV incidence, schistosomiasis, other infectious diseases [3]. We have previously shown an association between schistosomiasis infection and KSHV seropositivity and antibody levels in these communities [3]. These factors may explain the high risk of KSHV infection in this cohort. The rural GPC is a relatively stable population but has a high KSHV seropositivity probably due to factors such as frequent malaria infections (many asymptomatic), which could modulate the immune system impacting on KSHV viral control and transmission.

In conclusion, KSHV prevalence varies even within geographically proximate locations in Uganda. This variations could be caused by a number of co-factors including malaria, HIV and helminths infections and early age of KSHV infection. The high KSHV prevalence in various populations may be explained by the multiple co-factors that affect KSHV acquisition and transmission.

## Data Availability

Data can be made available on request.
